# A Multifunctional Antibacterial and Osteogenic Nanomedicine: QAS-Modified Core-Shell Mesoporous Silica Containing Ag Nanoparticles

**DOI:** 10.1155/2020/4567049

**Published:** 2020-09-19

**Authors:** Dexiong Li, Yubei Qiu, Sihui Zhang, Mi Zhang, Zexi Chen, Jiang Chen

**Affiliations:** ^1^School and Hospital of Stomatology, Fujian Medical University, Fuzhou, Fujian 350000, China; ^2^Stomatological Key Laboratory of Fujian College and University, Fujian Medical University, Fuzhou, 350002 Fujian, China; ^3^Institute of Stomatology & Research Center of Dental and Craniofacial Implants, School and Hospital of Stomatology, Fujian Medical University, Fuzhou, 350002 Fujian, China

## Abstract

Treatments for infectious bone defects such as periodontitis require antibacterial and osteogenic differentiation capabilities. Nanotechnology has prompted the development of multifunctional material. In this research, we aim to synthesize a nanoparticle that can eliminate periodontal pathogenic microorganisms and simultaneously stimulate new bone tissue regeneration and mineralization. QAS-modified core-shell mesoporous silica containing Ag nanoparticles (Ag@QHMS) was successfully synthesized through the classic hydrothermal method and surface quaternary ammonium salt functionalization. The Ag@QHMS in vitro antibacterial activity was explored via coculture with *Staphylococcus aureus*, *Escherichia coli*, and *Porphyromonas gingivalis* biofilms. Bone mesenchymal stem cells (BMSCs) were selected for observing cytotoxicity, apoptosis, and osteogenic differentiation. Ag@QHMS showed a good sustained release profile of Ag^+^ and a QAS-grafted mesoporous structure. Compared with the single-contact antibacterial activity of QHMS, Ag@QHMS exhibited a more efficient and stable concentration-dependent antimicrobial efficacy; the minimum inhibitory concentration was within 100 *μ*g/ml, which was below the BMSC biocompatibility concentration (200 *μ*g/ml). Thus, apoptosis would not occur while promoting the increased expression of osteogenic-associated factors, such as runt-related transcription factor 2 (RUNX2), alkaline phosphatase (ALP), osteopontin (OPN), osteocalcin (OCN), bone sialoprotein (BSP), and collagen type 1 (COL-1). A safe concentration of particles can stimulate cell alkaline phosphatase and matrix calcium salt deposition. The dual antibacterial effect from the direct contact killing of QAS and the sustained release of Ag nanoparticles, along with the Ag-promoted osteogenic differentiation, had been verified and utilized in Ag@QHMS. This system demonstrates the potential for utilizing pluripotent biomaterials to treat complex lesions.

## 1. Introduction

Clinically, periodontal diseases such as periodontitis, which leads to periodontal tissue lesions such as loose teeth, alveolar bone resorption, and even a variety of systemic diseases, are caused by the colonization and reproduction of pathogenic bacteria [1, 2]. At present, the main treatments for periodontitis include periodontal surgeries, such as implanting biocompatible anti-inflammatory materials, which have been reported to be beneficial for inhibiting the formation of plaque biofilms and promoting the repair of infectious bone defects [3, 4]. Therefore, the development of multifunctional composite materials has two main goals. On the one hand, the material should have a certain antibacterial effect to quickly eliminate factors that initiate periodontitis. On the other hand, the composite material should be able to regulate and promote periodontal tissue regeneration, such as alveolar bone regeneration, which has received increasing attention and may be used in a broad range of applications [5, 6].

Auxiliary materials that have been used in the clinical treatment of periodontitis mainly contain antibiotics, such as minocycline hydrochloride, and can produce relatively satisfactory anti-inflammatory effects. However, long-term antibiotic use will undoubtedly lead to an increase in drug resistance, thereby resulting in decreased efficacy [7]. Moreover, research has found that all antibiotics except low-dose doxycycline can reduce the expression of alkaline phosphatase in osteoblasts [8]. Therefore, there is an urgent demand to find novel antimicrobial materials to replace the currently used antibiotics. When in direct contact with bacterial cells, quaternary ammonium salts (QAS) with long alkyl chains can change the permeability of the cell membrane by altering its potential, thereby killing the bacteria; thus, QAS can kill bacteria without causing an increase in drug resistance [9, 10]. Our team successfully synthesized a dual-mode hollow mesoporous silica (HMS) carrier with both direct contact antibacterial properties and sustained drug eluting properties through a covalent modification with [3-(trimethoxysilyl) propyl] ammonium chloride [11]. This surface modification technology also reduces the cytotoxicity of nanoparticles, thus promoting the use of these materials in more applications. However, the above material lacks the ability to resist *Porphyromonas gingivalis*, which is the main pathogen of periodontitis. Thus, the currently designed dual antibacterial sustained nanoparticle release system needs further modification.

Silver nanoparticles demonstrate incomparable antibacterial effects and promote osteogenic differentiation; therefore, they are candidates for multifunctional systems [12, 13]. Studies have shown that 20~50 nm of silver nanoparticles with concentrations up to 2% can effectively inhibit microorganisms without causing obvious cytotoxicity [14]. Xie et al. [15] found that due to the accumulation of intracellular Ag nanoparticles, their cytotoxicity often appears to be time-dependent after being cocultured for many days. Furthermore, studies have shown that silver nanoparticles have an effect on osteogenic activity [16, 17]. Qin et al. [18] found that 4 *μ*g/ml of silver nanoparticles showed significantly higher osteogenic ability than pure Ag^+^. Similarly, the research results of Mahmood and colleagues showed that the expression level of osteoblasts such as ALP is promoted by the stimulation of nanomaterials containing silver nanoparticles and showed better osteoblast mineralization performance [19]. These results have prompted a large number of recent studies to look again at the application of silver nanoparticles, of which the development of effective sustained release systems for silver nanoparticles is most promising [20, 21].

In the present study, we hypothesize and successfully report that a multifunctional mesoporous silicon sustained drug release system can be synthesized by combining silver nanoparticles with a dual-mode antibacterial mesoporous silica. This complementary “multifunctional drug system” has the bacteriostatic effect of direct contact with long-chain QAS and the slow-release effect of silver nanoparticles. This system can not only effectively eliminate a variety of pathogenic bacteria in the initial stage of periodontal pathogen growth and reproduction but also stimulate new bone tissue regeneration and mineralization.

## 2. Materials and Methods

### 2.1. Fabrication of Ag Nanoparticles Encapsulated with QHMS (Ag@QHMS)

Tetraethyl orthosilicate (TEOS, AR), ammonium aqueous solution (25~28%), and cetyltrimethylammonium bromide (CTAB, AR) were acquired from Sigma-Aldrich (Shanghai, China). Carboalkylammonium chloride (SiQAC, purity 65%), silver nitrate (AgNO_3_), sodium citrate dehydrates, phosphate-buffered saline (PBS), sodium bicarbonate (Na_2_CO_3_), and ethanol were acquired from Xilong Scientific Cooperation (Beijing, China).

**Figure 1:**
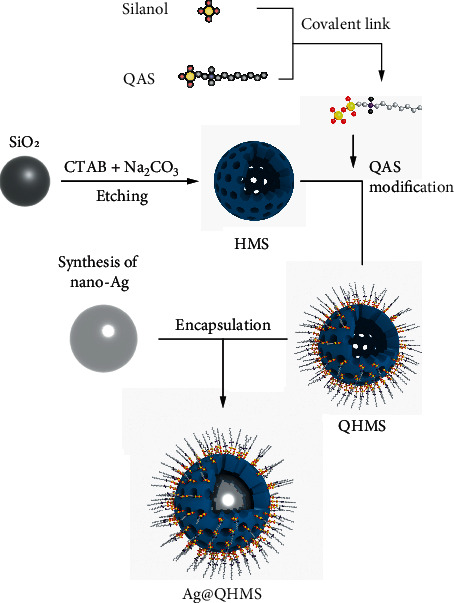
Synthesis procedure of HMS, QHMS, and Ag@QHMS.

The fabrication process is shown in Figure 1. In detail, silver nanoparticles were reduced by adding 11.5 mg sodium citrate dehydrate into 500 ml AgNO_3_ solution (18 mg/ml) at 100°C under severe agitation for 1 hour. The silver colloid was then centrifuged at 500 rpm for 1 hour to remove large nanoparticles after cooling down. Thereafter, 50 ml of the above colloid was diluted with 200 ml ethanol and the pH was adapted to 10. Next, 15 ml TEOS/ethanol (10 mM) was dripped into the mixture under constant severe agitation for 24 hours. Ag@SiO_2_ nanoparticles were collected through centrifugation at 8000 rpm for 10 minutes. Ultrasonically disperse Ag@SiO_2_ nanoparticles (20 mg) in 40 ml ethanol/water (3 : 5) solution, CTAB (75 mg), and ammonia solution (0.275 ml) for 30-minute stirring. Then, another 6 hours of stirring was performed by adding 1 ml TEOS : SiQAC (9 : 1) mixture. The product was collected and then etched with 2 mg/ml Na_2_CO_3_ solution under 60°C over 24 hours. The final powder was vacuum-dried after removing the surfactant CTAB through multiple ethanol extraction techniques. HMS and QHMS were synthesized as reported in the previous study [11].

### 2.2. Ag^+^ Effusion Profile in Simulated Body Fluid

Ag@QHMS (10 mg) was accurately dispersed in 50 ml of simulated body fluid with ultrasonic vibration for 15 minutes. The suspension was then constantly stirred at 1000 rpm for up to one month at 37°C. After 2, 6, 12, and 24 hours and 2, 3, 7, 10, and 15 days, the supernatant was collected by centrifugation at 8000 rpm for 10 minutes. The concentration of Ag^+^ in the supernatant was measured through inductively coupled plasma mass spectrometry (ICP-MS, Agilent Technologies, Beijing, China).

### 2.3. Material Characterization

Ag@QHMS was verified and analyzed by various characterization methods. The X-ray diffraction (XRD) spectra were obtained with a Bruker D8 Discover powder diffractometer (Brook Scientific, Beijing, China). Transmission electron microscopy (TEM) was conducted after ultrasonically dispersing the nanoparticles in ethanol and dropping them on a 200-mesh carbon film copper net to dry naturally. The sample was observed at 200 kV using a JEM-2100 microscope (JEOL Co., Ltd., Tokyo, Japan). In the meanwhile, the presence and content of Ag were measured with an Energy-Dispersive Spectrometer (EDS). Nitrogen adsorption-desorption isotherms were obtained with an ASAP2460 system (Micromeritics Instrument Co., Shanghai, China). Fourier transform infrared (FTIR) spectra were measured under a Nicolet is50 (Thermo Fisher Scientific, Shanghai, China) to detect peak shifts after QAS grafting.

### 2.4. Evaluation of Antibacterial Activity

#### 2.4.1. Minimum Inhibitory Concentration (MIC) and Minimum Bactericidal Concentration (MBC)

*Staphylococcus aureus* (ATCC 25923), *Escherichia coli* (ATCC 25922), and *Porphyromonas gingivalis* (ATCC 33277) were purchased from the American Type Culture Collection. The antimicrobial activity of Ag@QHMS was evaluated using MIC and MBC. Certain amounts of powers were suspended in 5 ml of Luria-Bertani liquid medium and diluted twice to obtain the final solution concentrations of 800, 400, 200, 100, 50, 25, 12.5, and 6.25. Then, these suspensions were cocultured with 5 × 10^5^ CFU/ml bacteria at 37°C for 24 hours. A positive control of the simple bacterial solution was also provided. After 24 hours of incubation, the growth of bacteria was observed and the turbidity was compared with that of the positive control group. The minimum concentration in the clear tube is the MIC of Ag@QHMS. The medium in the clear tube was then inoculated onto the solid medium plate. After incubation for 24 hours, the minimum antibacterial concentration without any bacterial growth was identified as the MBC.

#### 2.4.2. CFU Counting Experiment

In 24-well plates, the bacteria were cultivated and seeded (approximately 2 × 10^7^ colony-forming units (CFU)/ml) on cell climbing pieces to form mature single-strain biofilms. The Ag@QHMS suspension and biofilm were cocultured for 24, 72, and 168 hours. Then, at each time point, the sample was taken out and immersed in 5 ml of PBS and the biofilm was sufficiently separated by ultrasound. 10 *μ*l of the above microbial PBS mixture was reincubated on an agar plate for CFU counting. A simple biofilm without material served as a negative control, and 200 *μ*g/ml of HMS and QHMS and 25~200 *μ*g/ml of Ag@QHMS were used as the experimental groups. The antibacterial rate was calculated using the following formula:

(1) $$\begin{array}{c} \text{Antibacterial rate}\left(\%\right)=\frac{A-B}{A}\times 100\%, \end{array}$$

where *A* is the CFU in the negative control and *B* is the CFU in the experimental group.

#### 2.4.3. XTT Cell Viability Experiment

Each biofilm eluent was assayed using an XTT kit (Biological Industries, Kibbutz Beit Haemek, Israel). The residual mitochondrial dehydrogenase produced by living microbes can catalyse 3,3′-[1-(phenylamino)-3,4-tetrazolium]-bis (4-methoxy-6-nitro sodium benzene sulfonate) (XTT). The optical density (OD) of each biofilm was measured at 495 nm using an UV-Vis spectrophotometer (Metash Instruments Co., Shanghai, China).

#### 2.4.4. Fluorescence Staining of Biofilms

A single biofilm exposed under different concentrations of the Ag@QHMS suspensions was stained with the LIVE/DEAD BacLight™ Bacterial Viability Kit (Invitrogen, Thermo Fisher Scientific Inc., Shanghai, China) to identify the viability of the bacterial components in the biofilm. Bacteria with intact cell membranes can only be stained green by SYTO-9, while bacteria killed by cell membrane destruction are stained red by propidium iodide. A confocal scanning laser microscope (Carl Zeiss AG, Oberkochen, Germany) was used to observe the biofilm staining at 60x magnification. The excitation wavelength of SYTO-9 is 488 nm, and that of propidium iodide is 568 nm. For each type of biofilm, three sets of *Z*-stack images with an interval of 2 *μ*m were obtained.

### 2.5. Evaluation of Osteogenic Effects

#### 2.5.1. Cytotoxicity and Cell Proliferation

Bone mesenchymal stem cells (BMSCs, Chinese Scientific Faculty, Shanghai, China) of Sprague Dawley rat were cultured in low-glucose DMEM (HyClone, Thermal Scientific Co., USA) under 5% CO_2_ at 37°C to estimate the osteogenic effects. Briefly, 40 mg of HMS, QHMS, and Ag@QHMS nanoparticles was accurately weighed and suspended in 45 ml of DMEM medium and ultrasonically suspended for 10 minutes. Then, the suspension was incubated for 24 hours. Next, 5 ml of fetal bovine serum was added to form the particle-containing suspension of 800 *μ*g/ml and prepared for further dilution.

BMSCs (approximately 4 × 10^3^ cells) were seeded with DMEM in a 96-well plate. After cell adherence, the medium was substituted for one of the different concentrations of particle-containing suspensions. When cocultured for 2 days, a LDH cytotoxicity detection kit (Dojindo Laboratories, Shanghai, China) was introduced to quantitatively measure the cell viability after entrapping in different concentrations of the Ag@QHMS suspension. The absorbance was measured at the wavelength of 490 nm with a microplate reader (Shimadzu, Kyoto, Japan). The cytotoxicity rate was evaluated using the formula in Equation (2). Subsequently, the cell proliferation rate will be accessed with a Cell Counting Kit (Dojindo Laboratories, Shanghai, China) via the absorbance measured at 450 nm.

(2) $$\begin{array}{c} \text{Cytotoxicity}\left(\%\right)=\frac{B-A}{A}\times 100\%, \end{array}$$

where *A* is the absorbance of the negative control and *B* is the absorbance of the experimental group.

#### 2.5.2. Apoptosis

BMSCs (approximately 10^6^ cells) were cultured with DMEM in a 6-well plate. After incubating overnight for cell adherence, the original DMEM was substituted with the suspension that contained the same concentration of HMS, QHMS, and Ag@QHMS for 72 hours. Subsequently, the medium was discarded and rinsed twice with cold PBS, which was followed by cell digestion with EDTA-free trypsin. It was then resuspended to a cell density of 10^5^ according to the instructions of the annexin V-PI apoptosis detection kit (Solarbio Science & Technology Co., Beijing, China) and then analyzed on an Accuri™ C6 flow cytometer (BD Bioscience, Shanghai, China).

#### 2.5.3. Gene Expression

Quantitative reverse transcription polymerase chain reaction (qRT-PCR) was introduced to further investigate the expression levels of runt-related transcription factor 2 (RUNX2), alkaline phosphatase (ALP), osteopontin (OPN), osteocalcin (OCN), bone sialoprotein (BSP), collagen type 1 (COL-1), and other osteogenic markers. TRIzol (Takara Biomedical Technology Co., Ltd., Beijing, China) was used to extract the total RNA of the cells after 14 days of culture, and reverse transcription was performed according to the PrimeScript™ RT reagent kit with gDNA Eraser (Takara Biomedical Technology Co., Ltd., Beijing, China) to generate the template, and it was then mixed with TB Green® Premix Ex Taq™ (Takara Biomedical Technology Co., Ltd., Beijing, China) for PCR on a LightCycler® 480 instrument (Roche Diagnostics Ltd., Shanghai, China). The target gene primer sequences are shown in Table 1. The resulting mRNA levels were normalized to GAPDH and compared with those of the control group using the 2^−*ΔΔ*ct^ method.

#### 2.5.4. Mineralization Experiment

The mineralization ability experiments include an alkaline phosphatase activity (ALP) test and BCIP/NBT alkaline phosphatase and Alizarin Red staining. The specific process was as follows: 5 × 10^4^ BMSCs were seeded per well in a 6-well plate, and after 7 days of culture, the cells were washed with PBS, lysed overnight with 0.1% Triton X-100 at 4°C, and then centrifuged at 3000 rpm for 5 minutes to collect the supernatant. The ALP activity was evaluated by measuring the absorbance at 405 nm to detect the concentration of p-nitrophenyl phosphate (p-NPP) in the cell lysate through the alkaline phosphatase assay kit (Solarbio Science & Technology Co., Beijing, China). At the same time, cells were stained for ALP using the BCIP/NBT alkaline phosphatase color kit (Beyotime, Beijing, China) after 7days. After washing the cells twice with PBS, they were fixed in 4.0% paraformaldehyde on ice for 30 minutes. After washing 3 times with PBS, the cells were stained with 0.33% BCIP/0.66% NBT buffer for 10 minutes. The stained sample was observed and imaged through a microscope at 4x magnification. The matrix mineralization deposit was assessed by Alizarin Red staining after 2 weeks. The cells were fixed in 4.0% paraformaldehyde and stained with 1 ml of 1% Alizarin Red solution (Solarbio Science & Technology Co., Beijing, China) for 30 minutes. The staining solution was completely removed, and the cells were washed thoroughly with ddH_2_O. The stained sample was observed and imaged.

### 2.6. Statistical Analysis

Statistical analysis was performed by using SPSS Statistics 22.0 (IBM Co., Chicago, IL, USA). The normality and equal variance distribution of each data were analyzed using the Shapiro-Wilk and Levene tests, respectively. Thereafter, the data were analyzed via one-way analysis of variance and the Bonferroni pairwise comparisons. Statistical significance was preset at an alpha value of 0.05.

## 3. Results

### 3.1. Material Characterization

**Figure 2:**
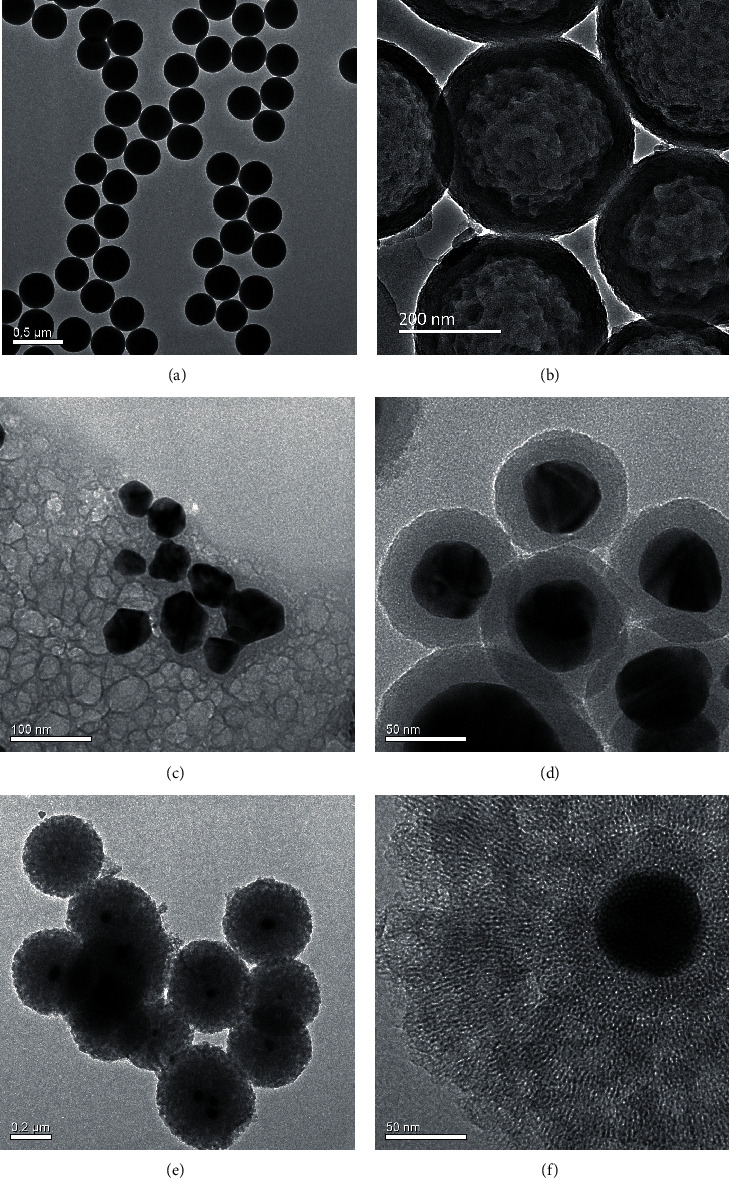
TEM images of the (a) SiO_2_ sphere, (b) hollow mesoporous silica, (c) silver nanoparticles, (d) silver nanoparticles coated with SiO_2_, (e) mesoporous Ag@QHMS, and (f) high-magnification view of the mesoporous structure.

TEM images (Figure 2) showed the morphology and structure of the main products during the synthesis of Ag@QHMS. Namely, Figure 2(a) shows the silicon sphere of approximately 250 nm, which was prepared for etching of the hollow QHMS (Figure 2(b)); Figure 2(c) shows the reduction of silver nanoparticles with a diameter of approximately 20~50 nm; Figure 2(d) shows the encapsulation of the mesoporous SiO_2_ shell; and Figure 2(e) shows the final complete mesoporous structure of Ag@QHMS. The 220~250 nm Ag@QHMS displayed the typical structure with an Ag-core mesoporous silica shell nanoparticle under the high-magnification sectional view (Figure 2(f)).

**Figure 3:**
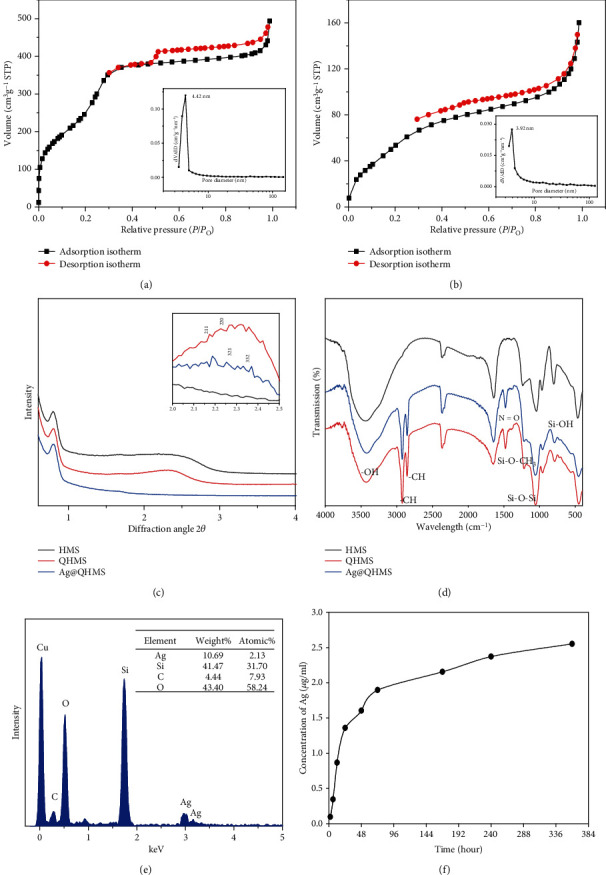
Characterization of HMS, QHMS, and Ag@QHMS: (a) nitrogen sorption and desorption isotherms of QHMS; (b) nitrogen adsorption and desorption isotherms of Ag@QHMS; (c) X-ray diffraction spectra; (d) infrared spectra; (e) EDS spectra of Ag@QHMS; (f) Ag effusion profile in simulated body fluid.

The nitrogen absorption-desorption isotherms of QHMS (Figure 3(a)) and Ag@QHMS (Figure 3(b)) were characteristic of the type IV mesoporous structure. More detailed data calculated by the BET and BJH models are shown in Table 2. Due to the fact that the hollow cavity was occupied by silver nanoparticles, the specific surface area of Ag@QHMS decreases significantly without changes in the mesopore diameter.

The small-angle XRD of HMS yielded characteristic peaks of the mesoporous structure, and the intensity decreased after QAS modification (QHMS) and the encapsulation of Ag (Ag@QHMS) (Figure 3(c)). Infrared absorbance spectra (Figure 3(d)) also displayed peak shifts among HMS, QHMS, and Ag@QHMS. The spectrum of HMS showed the characteristic peak of pure silica. The characteristic peaks of the Si-O-Si bonds were detected at approximately 462, 798, and 1066 cm^−1^, respectively. At 920~972 cm^−1^, a small-frequency band was marked as Si-OH vibration, and at 3000~3750 cm^−1^, a wide-frequency band was marked as O-H vibration. The peak at 1033~1151 cm^−1^ was attributed to Si-O-Si stretching, while the melting peak at 1220 cm^−1^ to the left was assigned to the Si-OCH_3_. The small peak at 967 cm^−1^ was associated with the vibration of Si-OH. These peaks coexist with Si-O-Si and Si-OH in HMS but cannot be clearly distinguished from QHMS after modification with QAS. The two larger peaks around 2858 cm^−1^ and 2927 cm^−1^ belonged to the -CH tensile vibration of QAS. Peaks at 1345~1555 cm^−1^ were marked as N=O vibrations. Although these peaks are not unique to QAS, they were not detected in HMS, which indicates the QAS functionalization of QHMS and Ag@QHMS.

Energy-dispersive spectra showed the main element content in Ag@QHMS indicating the successful encapsulation of Ag accounting for about 10% (Figure 3(e)). The content of C in mesoporous silica was attributed to the alkyl groups of functionalized QAS.

### 3.2. Ag^+^ Effusion Profile

The biological impacts of Ag nanoparticles are usually thought to be affected by Ag^+^ released after contact with water. The volume of Ag^+^ effused from Ag@QHMS, as measured by ICP-MS in the simulated body fluid (Figure 3(f)), showed a burst release of Ag^+^ over 24 hours and tended to show a stable and slow release within the following 15 days. This trend also indicates the long-term presence of the nanoparticles.

### 3.3. Antimicrobial Activity

*E. coli* (gram-negative bacteria), *S. aureus* (gram-positive bacteria), and *P. gingivalis* (characteristic pathogens in oral diseases) were utilized to estimate the antibacterial efficiency of Ag@QHMS nanoparticles. MIC experiments of these three strains were studied. Compared to the negative control, the Ag@QHMS groups exhibited better antimicrobial effect. The proliferation rates of *E. coli*, *S. aureus*, and *P. gingivalis* were clearly constrained by Ag@QHMS, whose MIC values were 50 *μ*g/ml, 100 *μ*g/ml, and 50 *μ*g/ml, respectively, and MBC values were 100 *μ*g/ml, 200 *μ*g/ml, and 100 *μ*g/ml, respectively. Obvious gram selectivity was not observed due to the broad-spectrum antibacterial properties of the nanoparticles.

**Figure 4:**
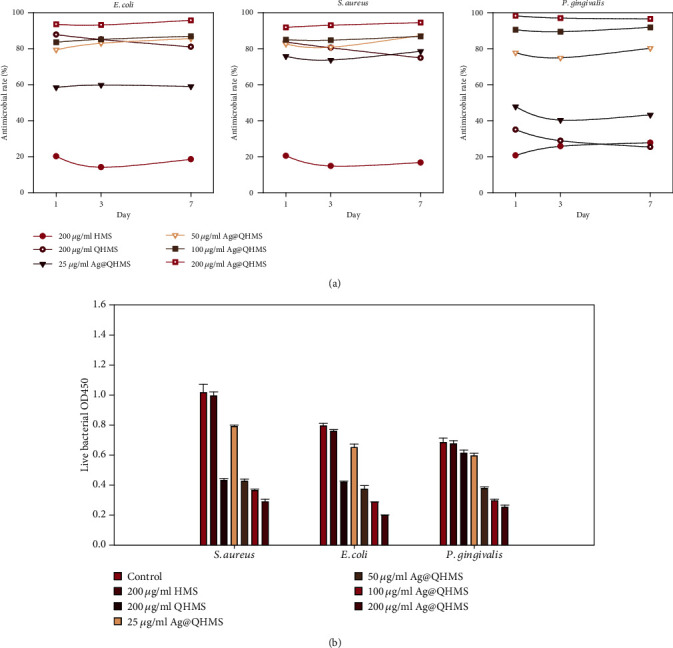
Antibacterial activity assessments: (a) CFU evaluation of the antimicrobial efficacy against three strains for 1 week and (b) bacterial viability measured by the XTT assay after 72 hours.

The antimicrobial effects of HMS, QHMS, or 25~200 *μ*g/ml Ag@QHMS on *E. coli*, *S. aureus*, and *P. gingivalis* were measured by the CFU counting method (Figure 4(a)). Regarding *E. coli* and *S. aureus*, the materials showed the same trend regarding the concentration-dependent antibacterial effect, that is, 200 *μ*g/ml Ag@QHMS > 100 *μ*g/ml Ag@QHMS ≈ QHMS > 50 *μ*g/ml Ag@QHMS > 25 *μ*g/ml Ag@QHMS > HMS > control (*p* < 0.05). Compared with the same concentration of HMS and the control group, pure QHMS effectively eradicated the *E. coli* and *S. aureus* biofilms; however, its inhibition effect on *P. gingivalis* was significantly weaker than that of the low concentration of Ag@QHMS with Ag nanoparticles.

The XTT analysis of the survival rate of bacteria for 3 days after reaction to the three strains of biofilms is shown in Figure 4(b). With the increase in Ag@QHMS concentration, the survival rate of bacteria decreased significantly and the effect in response to the three kinds of bacteria was similar to the results obtained from CFU counting. QHMS had a significant elimination effect on *S. aureus* and *E. coli*, although it was not particularly effective on *P. gingivalis*, whose efficiency was equivalent to that of Ag@QHMS at 50 *μ*g/ml.

**Figure 5:**
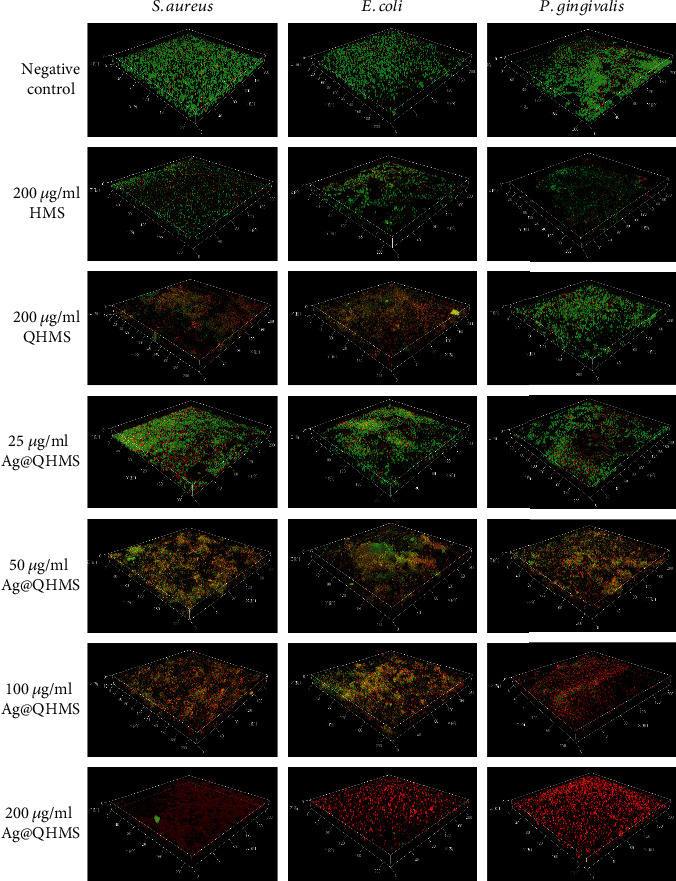
Confocal laser scan microscope images of single-species bacterial biofilm after coculture with HMS, QHMS, and 25~200 *μ*g/ml Ag@QHMS through Live/Dead BacLight stain after 3 days. Green fluorescence: live microbes. Red fluorescence: dead microbes.

Antimicrobial ability was further studied by laser confocal scanning microscopy with the living dead bacterial staining within 72 hours. The reconstructed image of the laser confocal microscope is shown in Figure 5, in which red represents dead bacteria and green represents living bacteria. These images show that QHMS grafted with QAS had a better antibacterial effect than the same concentration of HMS in terms of *E. coli* and *S. aureus* while QHMS presented little effect on eliminating the biofilm of *P. gingivalis*. However, 100~200 *μ*g/ml Ag@QHMS was obviously more effective in eliminating the biofilms we all tested.

### 3.4. Cytotoxicity and Cell Proliferation

**Figure 6:**
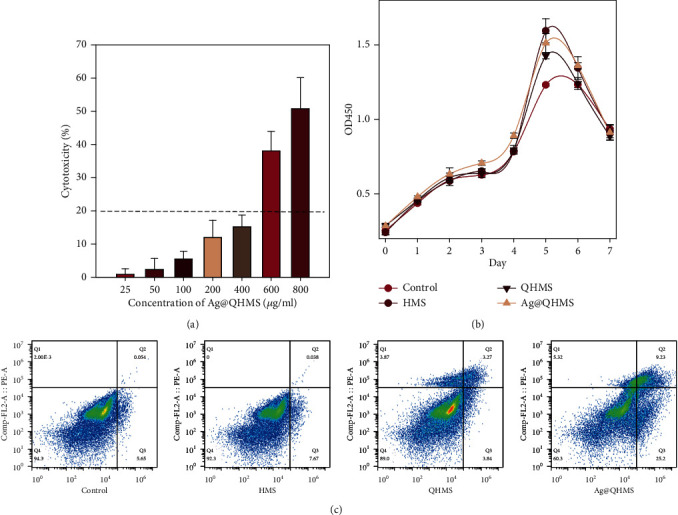
Cytotoxicity and apoptosis: (a) LDH cytotoxicity of different concentrations of Ag@QHMS; (b) proliferation rate of BMSCs cocultured with 200 *μ*g/ml of HMS, QHMS, and Ag@QHMS for 1 week; (c) apoptosis of BMSCs exposed to 200 *μ*g/ml of HMS, QHMS, and Ag@QHMS for 3 days.

Cleavage of the tetrazolium salt WST to formazan is proportional to the NADH produced by lactate dehydrogenase (LDH) which reduced nicotinamide adenine dinucleotide phosphate from inactive cells. Hence, the absorbance intensity of formazan can be an indirect reflection of the quantity of viable cells [22]. The cell damage rate results for the BMSCs exposed to different concentrations of Ag@QHMS over 2 days are shown in Figure 6(a). With increasing material concentration, the cell damage rate correspondingly increased. Compared with the control group, the average cell damage at concentrations below 400 *μ*g/ml was less than 20%, which is considered to be the lower limit of cytotoxicity. Moreover, in the 800 *μ*g/ml group, the cytotoxicity increased sharply to 50.93%, showing a significant statistical difference. However, Figure 6(b) shows that Ag@QHMS within the safe concentration range of 200 *μ*g/ml, which is consistent with that of HMS and QHMS, slightly promoted cell proliferation at 5 days. And this effect was only maintained until the 6th day; that is, it fell back to the trend of the control group.

### 3.5. Apoptosis

Annexin V can be used as a sensitive indicator to reflect early apoptosis due to its high affinity for phosphatidylserine, which is often exposed on cells in early apoptosis. It can be distinguished from propidium iodide, which can only penetrate midlate apoptotic or dead cells with increased membrane permeability. Our results (Figure 6(c)) showed that the apoptosis rate with HMS was equivalent to that of the control group. The apoptotic cell ratio in the QHMS group (3.27%) was slightly higher than that in the HMS group, whereas the apoptotic cell ratio in the Ag@QHMS group was highest at up to 9.23%, which may have been related to the addition of Ag particles.

### 3.6. Gene Expression

**Figure 7:**
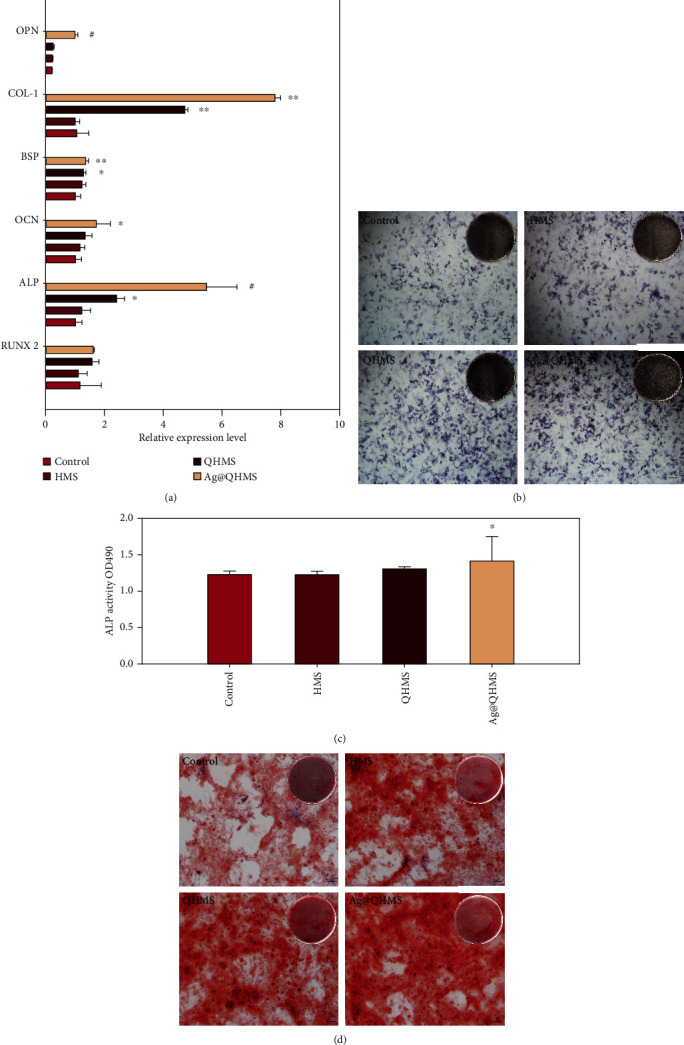
Osteogenic activity assessments. (a) Relative expressions of OPN, RUNX2, ALP, OCN, COL-1, and BSP in BMSCs after 2 weeks. The resulting mRNA levels were normalized to GAPDH and compared with those of the control group. ^∗^*p* < 0.05, ^∗∗^*p* < 0.01, and ^#^*p* < 0.001). (b) ALP staining of BMSCs after 1 week. (c) ALP activity of BMSCs after 1 week. (d) Alizarin Red staining after 2 weeks.

RUNX2, ALP, OPN, OCN, BSP, and COL-1 are well-known osteogenic markers, and their mRNA expression levels were elevated significantly as shown in Figure 7(a). Furthermore, differences in OCN, OPN, and RUNX2 expression were not observed among the QHMS, HMS, and control groups. QHMS slightly enhanced the expression of BSP and ALP compared to the HMS and control groups (*p* < 0.05) but showed a significant difference in COL-1 expression compared with these groups (*p* < 0.01). In contrast, Ag@QHMS promoted calcium deposition and the expression in all groups after 2 weeks. Among these markers, the expression of early factors, such as RUNX2 and BSP, did not show large changes while the expression of OPN, ALP (*p* < 0.001), and COL-1 (*p* < 0.01) increased considerably because of their accumulation over the entire osteogenesis process.

### 3.7. Mineralization Experiment

Alkaline phosphatase staining and matrix mineralization deposition are the most common indicators used to determine osteogenic activity. As shown directly in Figure 7(b) and the statistical results in Figure 7(c), HMS and QHMS seem to also increase the expression of ALP, although only the increase in ALP in the Ag@QHMS group was statistically significant (*p* < 0.05). The same trend also appeared for Alizarin Red staining after 14 days (Figure 7(d)), and more mineralized deposits were also found in the Ag@QHMS group. These data demonstrate that Ag@QHMS containing nanosilver particles has better osteoinductive activity than pure HMS and QHMS.

## 4. Discussion

Recently, most implant materials used in clinical practice are basically single-use, and most of the systems studied in the past have loaded only one drug; these systems can only achieve efficient but simple antibacterial or osteogenic support [4]. However, due to the limited nature and complexity of the lesion or repair area, there is an urgent need for efficient multifunctional materials. The development of nanotechnology has made it possible to design effective multifunctional antibacterial nanomaterials for multipurpose biological applications [23, 24]. HMS nanoparticles, due to their superior loading capacity and surface silanol functional groups, can be grafted for functionalization and good biodegradability, making them potential candidates in the development of multifunctional sustained release systems [25–27]. Previous published research showed that the HMS system can be used as a carrier with a certain antibacterial efficacy by covalently anchoring long-chain QAS with the active silanol functional groups on HMS; additionally, the material contains porous channels and an inner core cavity. Thus, the above construction can be used as a carrier or wrap for drugs or materials and achieve multifunctionality.

Plaque biofilms are known to be the initiating factors for oral diseases such as periodontitis or peri-implantitis, which cause infections and eventually alveolar bone defects [28]. At present, basic clinical treatment can effectively block the progress of infection, but improving bone restoration in the infected area must rely on the implantation of effective bone grafting materials to retain the three-dimensional space needed for new bone formation [29, 30]. More than 80% of microbial infections are associated with biofilm formation [31]. The key to solving biofilm formation onto the surface of biological materials is to control the initial adhesion of bacteria [32]. Therefore, giving the biomaterial surface inherent contact antibacterial properties appears to represent a more effective strategy for counteracting the early colonization of bacteria than the release of local concentrations of antibiotic [33]. In this study, QHMS relied on the direct contact killing effect of the surface-modified QAS to effectively inhibit the proliferation of *S. aureus* and *E. coli* within 1 day. However, the direct contact antibacterial activity of QHMS is passive antibacterial activity [34]. In the initial stage, due to the existence of long-chain QAS on the surface, microorganisms that directly contact with the antibacterial surface are likely to die and cannot easily form microbial colonies; thus, the formation of biofilms is inhibited in the initial stage. However, with the growth in the volume of microorganisms, the microorganisms in the upper layer lost the opportunity to directly touch the antibacterial surface. Thus, these microorganisms survive and begin to proliferate, resulting in the antibacterial rate gradually decreasing over time as observed in the experimental results. Moreover, QAS lacks effectiveness against *P. gingivalis*, which is a shortcoming of QAS found in published research [11]. In the present work, we employ Ag nanoparticles, which are highly developed and broad-spectrum antibacterial agents, to synergistically supplement the antibacterial capability of this system [35]. Hence, Ag@QHMS demonstrates a more efficient antibacterial effect on all the three strains with a concentration-dependent relationship. At the same time, due to the synergistic broad-spectrum antibacterial activity, not only is the possible drug resistance crisis avoided [4, 36] but the concentration of mesoporous silicon can also be reduced to keep it fully within the safe range; therefore, the worry about the biological safety of Ag nanoparticles can also be eliminated.

In terms of the cytotoxicity, there may be two possible genetic reasons. On the one hand, as reported by Beck et al. [37], small-sized mesoporous silicon particles may promote the proliferation of osteoblasts. On the other hand, the low concentration of silver nanoparticles packaged in the hall can also effectively promote short-term cell proliferation [15]. For hollow porous silicon, the pore diameter and hollow area are considered to be important parameters to improve drug loading capacity and dominate the release kinetics [38]. The double-layer SiO_2_ shell has a better delayed release effect than the single-shell HMS because of the double-layer SiO_2_ shell having a longer drug diffusion distance and the barrier effect stemming from the small pores in the inner shell [39]. In the first 6 days, the QHMS and Ag@QHMS displayed a slightly positive influence on the proliferation of BMSCs. It is considered that the slowly released Si and Ag ions in solution may then affect the cytoskeleton through the ROCK-1 pathway mediated by *β*-integrin, which leads to the promotion of osteoblast division and differentiation [18, 40]; however, the specific mechanism still needs to be further verified. After 7 days of coculture, the cell proliferation in all groups decreased, although the difference was not significant compared with the negative control group. The toxicity of Ag^+^ is generally considered to depend on the long-term intracellular aggregation concentration [41]. In this paper, the cumulative release of Ag^+^ from Ag@QHMS did not exceed 4 *μ*g/ml Ag^+^ over the monitoring period, which is consistent with the conclusion that concentrations of Ag particles lower than 5 *μ*g/ml do not show clear cytotoxicity [42]. However, according to the proliferation and declining trend in the later stage, we should be very cautious about the biological use of a high Ag^+^ concentration.

Some studies have suggested that the antibacterial or damaging effects of silver nanoparticles on cells are the result of cell apoptosis caused by silver nanoparticles anchored to the membrane [43]. The results show that long-chain QAS grafting on the surface of HMS cannot effectively promote apoptosis, which indirectly supports QAS leading to cell death through leakage and autolysis. Although the release of silver nanoparticles results in more apoptosis to some extent, it cannot be ascertained that silver nanoparticles promote apoptosis-induced cell damage. For example, in Gahlawat et al.'s research [44], the apoptosis results were considered to be more related to the high specific surface area of the small-sized silver nanoparticles, which allowed them to release more silver ions that can easily penetrate the membrane. However, the Ag nanoparticles employed in our study were lager than10 *μ*m; thus, their release was hindered by the double mesoporous shell. These inconsistent conclusions require more in-depth research to explore the changes in apoptosis.

In addition, as the reported results are consistent, our results also indicate that the sustained low-concentration Ag nanoparticle release from Ag@QHMS may promote osteogenic effects. ALP is one of the phenotypic markers of osteoblasts, which can directly reflect the activity or functional status of osteoblasts. COL-1 and BSP are matrix components that are abundant in bone tissue. OCN occurs mainly during the period of mineralization and is considered to be one of the differentiation markers of osteoblasts for the stage of mineralization. OPN plays an important role in the process of osteoblast attachment and mineralization. RUNX2 plays an important role in osteoblast differentiation and bone matrix mineralization for participating in the regulation of the expression of the aforementioned factors. The expression levels of these abovementioned osteogenic markers were upregulated compared to those of the QHMS group after 14 days, indicating that this increase may be by the biological effect caused by the sustained release of Ag nanoparticles. Particularly, the large increase in ALP expression showed that a low concentration of Ag nanoparticles can promote the accumulation of calcium and phosphorus ions, which is an important process of bone mineralization. The results of alkaline phosphatase staining and matrix mineralization deposition, which are similar to the difference in transcription expression level, also indicate that the introduction of nanosilver makes the surface of silica or modification of quaternary ammonium salts, which do not have effective biological activity, appear to have a gratifying effect of promoting bone induction through upregulating osteogenic genes. These findings are consistent with the research by He et al. [45] but contradict those of Samberg et al. [46], in which Ag nanoparticles were of no avail on the bone-forming effects of fat stem cells. This difference may be explained in part by different observation periods. Samberg et al. studied cells coincubated under a low concentration of Ag nanoparticles over 24 hours. However, bone differentiation induction is usually a long-term process, so a 24-hour observation period may not be sufficient to detect changes in gene expression levels. In contrast, our study used the established 14 days to detect changes, and the results show significant changes.

Within our research, the excellent multifunctional performance of Ag@QHMS has been confirmed. The basic mechanism of silver on osteogenesis has not yet been explored and requires further research. Moreover, the application of mesoporous silicon nanoparticles combined with other media such as metals is still a major limitation for its use in future research prospects as a medical implant material. However, many recent studies have successfully used nanoparticles and osteogenic scaffold matrices to synthesize composite scaffolds; thus, there is inspiration for the clinical application of mesoporous silicon nanoparticles [47, 48]. Furthermore, we commit to studying the practical application of this nanomaterial.

## 5. Conclusions

A novel bifunctional mesoporous silica nanomedicine was easily and successfully synthesized for biomedical applications. The Ag@QHMS presents dual antibacterial effects of direct contact killing based on QAS modification and dose-dependent killing based on Ag^+^ sustained release. The osteogenic-promoting effects and safety of the concentration of 400 *μ*g/ml were effectively guaranteed. The excellent multifunctional performance of Ag@QHMS indicates the potential applicability of this material in the future.

## Figures and Tables

**Table 1:** Primers employed for qRT-PCR.

Factors	Forward primer sequence (5′-3′)	Reverse primer sequence (5′-3′)
RUNX2	TCCCAGTATGAGAGTAGGTGTCC	GGCTCAGATAAGAGGGGTAAGAC
ALP	CCTAGACACAAGCACTCCCACTA	GTCAGTCAGGTTGTTCCGATTC
COL-1	TCTGACTGGAAGAGCGGAGAG	GAGTGGGGAACACACAGGTCT
OCN	GACCCTCTCTCTGCTCACTCTG	CACCTTACTGCCCTCCTGCTT
BSP	CGGCCACGCTACTTTCTTTA	CCCTCCTCCTCCGAACTATC
OPN	CAGACACCACTGTAACCTAGAA	TTGCCTGCCTCTACATACATT
GAPDH	CGGCAAGTTCAACGGCACAGTCAAGG	ACGACATACTCAGCACCAGCATCACC

**Table 2:** Structural parameters of QHMS and Ag@QHMS obtained by BET and BJH.

Nanoparticle	Specific surface area	Total pore volume	Pore diameter
QHMS	896.58 m^2^ g^−1^	0.7635 cm^3^ g^−1^	4.42 nm
Ag@QHMS	251.82 m^2^ g^−1^	0.2478 cm^3^ g^−1^	3.92 nm

## Data Availability

The data used to support the findings of this study are available from the corresponding author upon request.

## References

[1] Batool F., Strub M., Petit C. (2018). Periodontal tissues, maxillary jaw bone, and tooth regeneration approaches: from animal models analyses to clinical applications. *Nanomaterials*.

[2] Pihlstrom B. L., Michalowicz B. S., Johnson N. W. (2005). Periodontal diseases. *The Lancet*.

[3] Xu X., Gu Z., Chen X. (2019). An injectable and thermosensitive hydrogel: promoting periodontal regeneration by controlled-release of aspirin and erythropoietin. *Acta Biomaterialia*.

[4] Lu H., Liu Y., Guo J., Wu H., Wang J., Wu G. (2016). Biomaterials with antibacterial and osteoinductive properties to repair infected bone defects. *International Journal of Molecular Sciences*.

[5] Thomas M. V., Puleo D. A. (2011). Infection, inflammation, and bone regeneration: a paradoxical relationship. *Journal of Dental Research*.

[6] He X. T., Li X., Xia Y. (2019). Building capacity for macrophage modulation and stem cell recruitment in high-stiffness hydrogels for complex periodontal regeneration: experimental studies in vitro and in rats. *Acta Biomaterialia*.

[7] Li J. Y., Wang X. J., Wang L. N. (2015). High in vitro antibacterial activity of Pac-525 against Porphyromonas gingivalis biofilms cultured on titanium. *BioMed Research International*.

[8] Park J. B. (2011). Effects of doxycycline, minocycline, and tetracycline on cell proliferation, differentiation, and protein expression in osteoprecursor cells. *The Journal of Craniofacial Surgery*.

[9] Gong S. Q., Niu L. N., Kemp L. K. (2012). Quaternary ammonium silane-functionalized, methacrylate resin composition with antimicrobial activities and self-repair potential. *Acta Biomaterialia*.

[10] Wei T., Zhan W., Cao L. (2016). Multifunctional and regenerable antibacterial surfaces fabricated by a universal strategy. *ACS Applied Materials & Interfaces*.

[11] Bai Y. M., Mao J., Li D. X. (2019). Bimodal antibacterial system based on quaternary ammonium silane-coupled core-shell hollow mesoporous silica. *Acta Biomaterialia*.

[12] Patil S., Singh N. (2019). Antibacterial silk fibroin scaffolds with green synthesized silver nanoparticles for osteoblast proliferation and human mesenchymal stem cell differentiation. *Colloids and Surfaces. B, Biointerfaces*.

[13] Zhou W., Jia Z., Xiong P. (2017). Bioinspired and biomimetic AgNPs/gentamicin-embedded silk fibroin coatings for robust antibacterial and osteogenetic applications. *ACS Applied Materials & Interfaces*.

[14] Swolana D., Kepa M., Idzik D. (2020). The antibacterial effect of silver nanoparticles on Staphylococcus epidermidis strains with different biofilm-forming ability. *Nanomaterials*.

[15] Xie H., Wang P., Wu J. (2019). Effect of exposure of osteoblast-like cells to low-dose silver nanoparticles: uptake, retention and osteogenic activity. *Artificial Cells, Nanomedicine, and Biotechnology*.

[16] Yan J., Zhou W., Jia Z. (2018). Endowing polyetheretherketone with synergistic bactericidal effects and improved osteogenic ability. *Acta Biomaterialia*.

[17] Croes M., Bakhshandeh S., van Hengel I. A. J. (2018). Antibacterial and immunogenic behavior of silver coatings on additively manufactured porous titanium. *Acta Biomaterialia*.

[18] Qin H., Zhu C., An Z. (2014). Silver nanoparticles promote osteogenic differentiation of human urine-derived stem cells at noncytotoxic concentrations. *International Journal of Nanomedicine*.

[19] Mahmood M., Li Z., Casciano D. (2011). Nanostructural materials increase mineralization in bone cells and affect gene expression through miRNA regulation. *Journal of Cellular and Molecular Medicine*.

[20] Zhang R., Lee P., Lui V. C. H. (2015). Silver nanoparticles promote osteogenesis of mesenchymal stem cells and improve bone fracture healing in osteogenesis mechanism mouse model. *Nanomedicine*.

[21] Toledano M., Gutierrez-Perez J. L., Gutierrez-Corrales A. (2020). Novel non-resorbable polymeric-nanostructured scaffolds for guided bone regeneration. *Clinical Oral Investigations*.

[22] Chan F. K.-M., Moriwaki K., de Rosa M. J. (2013). Detection of necrosis by release of lactate dehydrogenase activity. *Methods in Molecular Biology*.

[23] Pacheco H., Vedantham K., Aniket, Young A., Marriott I., el-Ghannam A. (2014). Tissue engineering scaffold for sequential release of vancomycin and rhBMP2 to treat bone infections. *Journal of Biomedical Materials Research. Part A*.

[24] Xu Z., Lai Y., Wu D. (2015). Antibacterial effects and biocompatibility of titania nanotubes with octenidine dihydrochloride/poly(lactic-co-glycolic acid). *BioMed Research International*.

[25] Li Z., Zhang Y., Feng N. (2019). Mesoporous silica nanoparticles: synthesis, classification, drug loading, pharmacokinetics, biocompatibility, and application in drug delivery. *Expert Opinion on Drug Delivery*.

[26] Vieira S., Vial S., Reis R. L., Oliveira J. M. (2017). Nanoparticles for bone tissue engineering. *Biotechnology Progress*.

[27] Wang Y., Zhao Q., Han N. (2015). Mesoporous silica nanoparticles in drug delivery and biomedical applications. *Nanomedicine*.

[28] Qi M., Li X., Sun X. (2019). Novel nanotechnology and near-infrared photodynamic therapy to kill periodontitis-related biofilm pathogens and protect the periodontium. *Dental Materials*.

[29] He B., Ou Y., Chen S. (2017). Designer bFGF-incorporated d-form self-assembly peptide nanofiber scaffolds to promote bone repair. *Materials Science & Engineering. C, Materials for Biological Applications*.

[30] Zhang Y., Zheng Z., Yu M. (2018). Using an engineered galvanic redox system to generate positive surface potentials that promote osteogenic functions. *ACS Applied Materials & Interfaces*.

[31] Salwiczek M., Qu Y., Gardiner J. (2014). Emerging rules for effective antimicrobial coatings. *Trends in Biotechnology*.

[32] Cwalina B., Dec W., Michalska J. K., Jaworska-Kik M., Student S. (2017). Initial stage of the biofilm formation on the NiTi and Ti6Al4V surface by the sulphur-oxidizing bacteria and sulphate-reducing bacteria. *Journal of Materials Science. Materials in Medicine*.

[33] Jiao Y., Niu L. N., Ma S., Li J., Tay F. R., Chen J. H. (2017). Quaternary ammonium-based biomedical materials: state-of-the-art, toxicological aspects and antimicrobial resistance. *Progress in Polymer Science*.

[34] Li F., Weir M. D., Chen J., Xu H. H. K. (2014). Effect of charge density of bonding agent containing a new quaternary ammonium methacrylate on antibacterial and bonding properties. *Dental Materials*.

[35] Carrouel F., Viennot S., Ottolenghi L., Gaillard C., Bourgeois D. (2020). Nanoparticles as anti-microbial, anti-inflammatory, and remineralizing agents in oral care cosmetics: a review of the current situation. *Nanomaterials*.

[36] al-Dhabi N., Mohammed Ghilan A. K., Arasu M. (2018). Characterization of silver nanomaterials derived from marine Streptomyces sp. Al-Dhabi-87 and its in vitro application against multidrug resistant and extended-spectrum beta-lactamase clinical pathogens. *Nanomaterials*.

[37] Beck G. R., Ha S. W., Camalier C. E. (2012). Bioactive silica-based nanoparticles stimulate bone-forming osteoblasts, suppress bone-resorbing osteoclasts, and enhance bone mineral density in vivo. *Nanomedicine*.

[38] Jia L., Shen J., Li Z. (2012). Successfully tailoring the pore size of mesoporous silica nanoparticles: exploitation of delivery systems for poorly water-soluble drugs. *International Journal of Pharmaceutics*.

[39] Chen Y., Chen H., Ma M. (2011). Double mesoporous silica shelled spherical/ellipsoidal nanostructures: synthesis and hydrophilic/hydrophobic anticancer drug delivery. *Journal of Materials Chemistry*.

[40] Xu Y., Zheng B., He J., Cui Z., Liu Y. (2019). Silver nanoparticles promote osteogenic differentiation of human periodontal ligament fibroblasts by regulating the RhoA-TAZ axis. *Cell Biology International*.

[41] Yin I. X., Zhang J., Zhao I. S., Mei M. L., Li Q., Chu C. H. (2020). The antibacterial mechanism of silver nanoparticles and its application in dentistry. *International Journal of Nanomedicine*.

[42] Greulich C., Kittler S., Epple M., Muhr G., Koller M. (2009). Studies on the biocompatibility and the interaction of silver nanoparticles with human mesenchymal stem cells (hMSCs). *Langenbeck's Archives of Surgery*.

[43] De Matteis V., Cascione M., Toma C. C., Leporatti S. (2018). Silver nanoparticles: synthetic routes, in vitro toxicity and theranostic applications for cancer disease. *Nanomaterials*.

[44] Gahlawat G., Shikha S., Chaddha B. S., Chaudhuri S. R., Mayilraj S., Choudhury A. R. (2016). Microbial glycolipoprotein-capped silver nanoparticles as emerging antibacterial agents against cholera. *Microbial Cell Factories*.

[45] He W., Zheng Y., Feng Q. (2020). Silver nanoparticles stimulate osteogenesis of human mesenchymal stem cells through activation of autophagy. *Nanomedicine*.

[46] Samberg M. E., Loboa E. G., Oldenburg S. J., Monteiro-Riviere N. A. (2012). Silver nanoparticles do not influence stem cell differentiation but cause minimal toxicity. *Nanomedicine*.

[47] Kapoor S., Kundu S. C. (2016). Silk protein-based hydrogels: promising advanced materials for biomedical applications. *Acta Biomaterialia*.

[48] Abbott R. D., Kimmerling E. P., Cairns D. M., Kaplan D. L. (2016). Silk as a biomaterial to support long-term three-dimensional tissue cultures. *ACS Applied Materials & Interfaces*.

